# Supplementary material for: Haploblocks contribute to parallel climate adaptation following global invasion of a cosmopolitan plant
Source: Nat Ecol Evol. 2025 Jul 8;9(8):1441–55. doi: 10.1038/s41559-025-02751-2 (PMC12328208; doi:10.1038/s41559-025-02751-2)
Supplement: Supplementary file 2 — Reporting Summary [file 41559_2025_2751_MOESM2_ESM.pdf]

nature portfolio

Corresponding author(s): Nicholas Kooyers
Last updated by author(s): Dec 5, 2024

Reporting Summary

Nature Portfolio wishes to improve the reproducibility of the work that we publish. This form provides structure for consistency and transparency in reporting. For further information on Nature Portfolio policies, see our Editorial Policies and the Editorial Policy Checklist.

Statistics

For all statistical analyses, confirm that the following items are present in the figure legend, table legend, main text, or Methods section.

n/a	Confirmed	
☐	☒	The exact sample size (*n*) for each experimental group/condition, given as a discrete number and unit of measurement
☐	☒	A statement on whether measurements were taken from distinct samples or whether the same sample was measured repeatedly
☐	☒	The statistical test(s) used AND whether they are one- or two-sided<br>*Only common tests should be described solely by name; describe more complex techniques in the Methods section.*
☐	☒	A description of all covariates tested
☐	☒	A description of any assumptions or corrections, such as tests of normality and adjustment for multiple comparisons
☐	☒	A full description of the statistical parameters including central tendency (e.g. means) or other basic estimates (e.g. regression coefficient) AND variation (e.g. standard deviation) or associated estimates of uncertainty (e.g. confidence intervals)
☐	☒	For null hypothesis testing, the test statistic (e.g. *F*, *t*, *r*) with confidence intervals, effect sizes, degrees of freedom and *P* value noted<br>*Give P values as exact values whenever suitable.*
☒	☐	For Bayesian analysis, information on the choice of priors and Markov chain Monte Carlo settings
☒	☐	For hierarchical and complex designs, identification of the appropriate level for tests and full reporting of outcomes
☐	☒	Estimates of effect sizes (e.g. Cohen's *d*, Pearson's *r*), indicating how they were calculated

Our web collection on statistics for biologists contains articles on many of the points above.

Software and code

Policy information about availability of computer code

Data collection: Climate data for sampling locations from the WorldClim dataset was accessed via the raster v3.6-26 package in R v4.2.2

Data analysis: All code from this manuscript is linked to a github repository (github.com/pbattlay/glue-invasions). Briefly, all low coverage whole genome sequences were trimmed, aligned, and processed using fastp v23.2, bwa mem v0.7.17, and samtools v1.10. Quality control on each bamfile was performed using Qualimap v2.2.2, Bamtools v2.5.1, and multiQC v1.14. Population genomic diversity, differentiation, and structure using genotype likelihood approaches implemented in ANGSD v0.929. R v4.2.2 was used for all statistical comparisons including comparisons of summary statistics between ranges, Mantel tests, and PERMANOVAs. EPOS was used to assess effective population size through time. NGSadmix was used to calculate admixture coefficients and PCAngsd was used to conduct principal component analysis. BayPass was used for contrast, outlier, and gene environmental association analyses. Local PCA analysis and haploblocks were determined using customize code based off of lostruct software. Linkage disequilibrium was assessed using ngsLD v1.2.0. Gene ontology analysis was conducted using topGO v2.50.0 in R v4.2.2.

RNAseq reads trimmed using fastp v023.4. Microbial RNA contamination was removed by aligning to a custom database using bowtie2 v2.5.1. A transcriptome was created using gffread v0.12.7. Read mapping and quantification was done using Salmon v1.10.2. Differential expression analysis was assessed using DESeq2. Custom permutation code was written in R v4.2.2.

For manuscripts utilizing custom algorithms or software that are central to the research but not yet described in published literature, software must be made available to editors and reviewers. We strongly encourage code deposition in a community repository (e.g. GitHub). See the Nature Portfolio guidelines for submitting code & software for further information.

Data

Policy information about availability of data

All manuscripts must include a data availability statement. This statement should provide the following information, where applicable:

- Accession codes, unique identifiers, or web links for publicly available datasets
- A description of any restrictions on data availability
- For clinical datasets or third party data, please ensure that the statement adheres to our policy

Low coverage whole genome sequences (fastq files) for all accessions can be found as .fq files in the NCBI SRA database (Bioprojects: PRJNA1081485, PRJNA1179961). Metadata and fitness data from the four-way common garden study can be found on Dryad: https://datadryad.org/stash/share/hBvXInXHWCab2P1kIGKWALwX-Mz9KM6TYLPn9LAySS4, and associated low coverage whole genome sequences are in the NCBI SRA database (Biooproject: PRJNA1098360). Raw fastq files from RNAseq expression experiment can be found in the NCBI SRA database (Bioproject: PRJNA1131002). All code from this manuscript is linked to a github repository (github.com/pbattlay/glue-invasions).

Research involving human participants, their data, or biological material

Policy information about studies with human participants or human data. See also policy information about sex, gender (identity/presentation), and sexual orientation and race, ethnicity and racism.

Reporting on sex and gender	Not applicable
Reporting on race, ethnicity, or other socially relevant groupings	Not applicable
Population characteristics	Not applicable
Recruitment	Not applicable
Ethics oversight	Not applicable

Note that full information on the approval of the study protocol must also be provided in the manuscript.

Field-specific reporting

Please select the one below that is the best fit for your research. If you are not sure, read the appropriate sections before making your selection.

☐ Life sciences ☐ Behavioural & social sciences ☒ Ecological, evolutionary & environmental sciences

For a reference copy of the document with all sections, see nature.com/documents/nr-reporting-summary-flat.pdf

Ecological, evolutionary & environmental sciences study design

All studies must disclose on these points even when the disclosure is negative.

Study description

We combine a population genomic analysis across worldwide populations of white clover (Trifolium repens L.) with genome-wide association analysis of fitness within native and introduced populations conducted within four field common garden plots (low and high latitude gardens in the European and North American ranges), and RNAseq analysis in a manipulative dry-down experiment.

Research sample

The population genomic dataset consists of 2660 samples including 2648 samples from six continents. The majority of these samples (2616) come from sampling around 50 cities. Additional samples come from sampling around 4 cities in Spain as well as 12 popular cultivars.

The field common garden experiments utilized seeds derived from 93 populations collected along latitudinal gradients across North America and Europe. Seeds from each population were originally pooled from >20 individuals collected >3m apart. Field seeds were planted in a refresher generation and then outcrossed within populations to produce the lines grown in each common garden. Sequence data was obtained from 656 total individuals from these gardens.

The manipulative dry-down experiment included 51 individuals coming from field seed from 14 of the populations collected across latitudinal gradients in North America and Europe.

Sampling strategy

For the population genomics from low coverage whole genome sequencing, samples sizes from each population ranged from 5-120. We included extensively sampled populations for better estimates of site frequency spectra and population-genomic statistics (31 populations; Ave. = 80.74, Std. = 17.7 individuals) and added additional cities with lower sampling that we deemed as important areas for understanding colonization history (19 cities; Ave. = 5.95). We chose a sequencing coverage of ~1X for efficient, accurate and precise estimation of our various summary statistics of interest based on published low coverage whole genome sequencing

simulations. We downsampled cities that were outliers for coverage.

We sequenced every individual that we could obtain DNA from the field common garden experiments. Of 2000 individuals originally planted, we only were able to obtain DNA from 656. The number of samples from each range (Europe: 190 individuals, North America: 465) is sufficient to detect loci with moderate effect sizes within genome-wide association studies. Sequencing coverage paralleled the population genomic analysis above to provide a compatible dataset for detecting haploblocks.

Our RNAseq experimental design included a control and a dry down treatment. In each treatment, we planted 3 lines from each of the 16 populations (4 population from the low and high latitude populations in the native (Europe) and introduced ranges (North American). Our final sample size for RNAseq included 1-3 individuals per population in each of the treatments due to poor germination. This design provides sufficient biological replication within latitudes, ranges, and across treatments to assess differential expression between each contrast.

Data collection

Data collection for 2616 samples for the population genomics was done through the Global Urban Evolution Network (GLUE) by fellow scientists across the world. The collections for this dataset were first reported in Santangelo et al. 2022 (DOI: 10.1126/science.abk0989). Samples for populations from Spain was done by Simon Innes in 2021. The collection of these samples are first reported in Innes et al. 2022 (DOI: 10.1111/evo.14514). Sequencing and bioinformatic analysis was completed by Brandon Hendrickson, Jonas Mendez-Reneau, Paul Battlay, James Santangelo, Jonathan Wilson, Aude E. Caizergues, and Nicholas Kooyers.

Data collection for field common gardens was completed in North American Gardens from March 2020 to September 2021. Work in the Lafayette common garden was led by Nevada King and Courtney Patterson. Work in the Toronto common garden was led by Lucas Albano. Data collection in the European common gardens was conducted from March 2021-September 2022. Work in the Montpellier common garden was led by Cyrille Violle and Francois Vasseur. Work in the Uppsala common garden was led by Adriana Puentes and Amelia Tudoran. Analysis was done by Lucas Albano, Brandon Hendrickson, and Nicholas Kooyers.

RNAseq experiments were conducted in growth chambers from January 2020 – April 2020 with data collection by Caitlyn Stamps and Courtney Patterson. RNA extraction and library construction was completed by Hunter Strickland and Paul Kim. Bioinformatic analysis was completed by Michael Foster, Brandon Hendrickson, and Nicholas Kooyers.

Timing and spatial scale

Population genomics samples are collected from worldwide populations as described above.

Seed generation and sourcing for the field common gardens is described above. Field common gardens in North American Gardens ran from March 2020 to September 2021, while the European common garden was conducted from March 2021-September 2022. Data collection was performed weekly, biweekly, monthly or annually depending on which phenotype was being assessed. Data collection was only performed during the growing season.

Samples used in the manipulative growth chamber experiment were obtained from natural population in Europe and North America as described above.

Data exclusions

For the population genomics analysis, we excluded 8 samples based on both low coverage (below 0.5x) and quality of sequence data. These samples are not included within any numbers reported in the manuscript (i.e. we started with 2669 samples)

We had sequence data from 656 samples from the field common garden experiments; however, we excluded samples that have very limited numbers of reads post-sequencing. We had sufficient coverage and quality to call genotypes of haploblocks for 586 individuals and sufficient coverage (>0.5X) to conduct GWAS analysis for 569 plants.

We excluded a single individual from the RNAseq analysis as this individual was much larger than the others at the beginning of the experiment. We excluded three other samples from RNAseq analysis that had low read counts. Our total sample size was 47 individuals.

Reproducibility

Given the nature of our experiments (worldwide population genomics sampling, multiyear field experiments), cost (manipulative experiment with RNAseq), or availability of additional seed stock (manipulative experiment with RNAseq), we did not replicate our experiments.

Randomization

Planting location was randomized within each common garden for the field common garden experiments. Location of pots were randomized within treatment in the manipulative growth chamber experiment.

Blinding

Researchers were blinded from knowing the associated metadata (i.e. population or range origin) while collecting data as each plant was given a plant ID based on the randomized location within gardens or treatment prior to planting.

Did the study involve field work? ☒ Yes ☐ No

Field work, collection and transport

Field conditions

Temperature and precipitation varied both seasonally and between growing seasons. Generally, temperatures were slightly higher than historic climatic norms. There were no extreme events that impacted our gardens.

Location	Coordinates of common garden locations are 42°32′40″ N, 79°39′38″ W (Mississauga, Ontario, Canada), 30°18′23″ N, 92°00′3 3″ W (Lafayette, Louisiana, USA), 59°49′08″ N, 17°38′49″ W (Uppsala, Sweden), and 43°38′16″ N, 3°51′43″ W (Montpellier, France).
Access & import/export	All collections were done with permission of the local authorities as described in Santangelo et al. 2022 (DOI: 10.1126/science.abk0989), Innes et al. 2022 (DOI: 10.1111/evo.14514) and Albano et al. 2024 (DOI: 10.1101/2024.09.03.611023). Importation of tissue and seed between the EU and Canada as well as between Canada and United States went through required phytosanitary pathways by Canadian Food Inspection Agency or/and the USDA.
Disturbance	Common gardens were conducted within field stations in grassy areas that already had substantial human disturbance as white clover grows within lawns and grasslands. Landscape tarps were removed following the common gardens.

Reporting for specific materials, systems and methods

We require information from authors about some types of materials, experimental systems and methods used in many studies. Here, indicate whether each material, system or method listed is relevant to your study. If you are not sure if a list item applies to your research, read the appropriate section before selecting a response.

Materials & experimental systems

n/a	Involved in the study	
☒	☐	Antibodies
☒	☐	Eukaryotic cell lines
☒	☐	Palaeontology and archaeology
☒	☐	Animals and other organisms
☒	☐	Clinical data
☒	☐	Dual use research of concern
☐	☒	Plants

Methods

n/a	Involved in the study	
☒	☐	ChIP-seq
☒	☐	Flow cytometry
☒	☐	MRI-based neuroimaging

Dual use research of concern

Policy information about dual use research of concern

Hazards

Could the accidental, deliberate or reckless misuse of agents or technologies generated in the work, or the application of information presented in the manuscript, pose a threat to:

No	Yes	
☒	☐	Public health
☒	☐	National security
☒	☐	Crops and/or livestock
☒	☐	Ecosystems
☒	☐	Any other significant area

Experiments of concern

Does the work involve any of these experiments of concern:

No	Yes	
☒	☐	Demonstrate how to render a vaccine ineffective
☒	☐	Confer resistance to therapeutically useful antibiotics or antiviral agents
☒	☐	Enhance the virulence of a pathogen or render a nonpathogen virulent
☒	☐	Increase transmissibility of a pathogen
☒	☐	Alter the host range of a pathogen
☒	☐	Enable evasion of diagnostic/detection modalities
☒	☐	Enable the weaponization of a biological agent or toxin
☒	☐	Any other potentially harmful combination of experiments and agents

Plants

Seed stocks

Collection locations and procedures for samples collected from the GLUE network are reported in Santangelo et al. 2022 (DOI: 10.1126/science.abk0989). Individuals were sampled from multiple urban and rural locations surrounding a focal city. Collection locations and procedures for the field common garden are described in), Innes et al. 2022 (DOI: 10.1111/evo.14514) and Albano et al. 2024 (DOI: 10.1101/2024.09.03.611023). Individuals were sampled from a single locality for each population in this work and field seed from each location was pooled from [illegible]20 individuals. Field seed was grown and outcrossed within populations in a greenhouse refresher generations. For both studies, care was taken to sample different genets in the field (i.e. sampling plant at least 3m apart).

Cultivar lines were obtained from the USDA GRIN database. Specific accessions were: PI 419973, PI 376882, PI 430569, NSL 5462, NSL 6517, NSL 4784, PI 631875, NSL 186524, PI 5459. Three other accessions (Durana, Patriot, and Renovation) were obtained from commercial horticulture distributors.

Novel plant genotypes

Not applicable

Authentication

Not applicable
